# An Electronic Disease Early Warning System in Sana’a Governorate, Yemen: Evaluation Study

**DOI:** 10.2196/14295

**Published:** 2019-11-19

**Authors:** Mona Mayad, Reema Alyusfi, Ali Assabri, Yousef Khader

**Affiliations:** 1 Yemen Field Epidemiology Training Program Administrator of Epidemiology Administration in National Center of Public Health Laboratories Sana'a Yemen; 2 Electronic Disease Early Warning System Electronic Disease Early Warning System, eDEWS National Coordinator, Sana’a, Yemen Sana'a Yemen; 3 Department of Public Health. Jordan University of Science & Technology Aman Jordan

**Keywords:** evaluation, eDEWS, field epidemiology, Yemen

## Abstract

**Background:**

Electronic Disease Early Warning System (eDEWS) is one of the effective programs in epidemiological surveillance.

**Objective:**

This study aimed to identify the strengths and weaknesses of eDEWS in Sana’a governorate, determine its usefulness, and assess its performance in terms of the system attributes, including simplicity, flexibility, data quality, acceptability, representativeness, timeliness, and stability.

**Methods:**

Updated guidelines on the evaluation of public health surveillance from the Center for Disease Control and Prevention (CDC) were used to evaluate the eDEWS in Sana’a governorate. Stakeholders from different levels were interviewed about the performance of the eDEWS.

**Results:**

The overall score for the usefulness of the eDEWS was good (mean=83%). The overall system performance was good (86%). The highest attribute score was 100% for representativeness and the lowest score was 70% for stability. The system simplicity and acceptability were good. Although the system representativeness and flexibility were excellent, the stability was average. System completeness and timeliness were 100%.

**Conclusions:**

In conclusion, eDEWS in Yemen is useful and met its main objective. The overall level of system performance was good.

## Introduction

### Background

Communicable diseases remain the most common cause of death, disability, and illness in many developing countries [1]. The most critical step to prevent and control epidemics effectively is the timely detection of outbreaks, which depends on effective disease surveillance systems.

Electronic Diseases Early Warning System (eDEWS) is an electronic system for data collection, compilation, and analysis from the health facilities to detect outbreaks at an early stage and take necessary response measures to prevent or limit disease occurrence. This can minimize the morbidity and mortality owing to communicable diseases through the detection of potential outbreaks at their earliest possible stage and facilitate timely interventions [2,3]. eDEWS is based on a mobile app. It enables health workers to report in real time about public health threats such as acute watery diarrhea, polio (Acute Flaccid Paralysis), measles, meningitis, and viral hemorrhagic fevers, including Ebola. Through eDEWS, data are collected using software installed on mobile phone, tablets, or laptops and immediately sent to a central level for analysis and rapid response. With faster reporting, increase in the number of patients with diseases of public health concern can be spotted more quickly, enabling prompt follow-up action [4].

Surveillance systems in most of the Eastern Mediterranean countries are traditional and still depend on manual recording, which causes delay in reporting and also missing data. In response to the need for strengthening the existing surveillance systems and improving the speed and efficiency of data collection, analysis, and public health response, World Health Organization (WHO) in collaboration with the Ministries of Health decided to implement the eDEWS in several countries [3,5].

eDEWS was launched in Yemen in March 2013, at 4 governorates, Aden, Lahij, Abyan, and Taiz, in 100 health facilities for 16 priority infectious diseases [5-7].

The various surveillance activities are integrated into 1 system within the broader national health information to enhance the speed and efficiency of data collection, analysis, and public health response [3].

Since the beginning of the War in March 2015, limited access to health care services and a breakdown in safe water supply and sanitation services has triggered the spread of endemic diseases such as malaria and dengue fever, as well as acute diarrheal diseases.

In response to the need for electronic expansion of the existing public health surveillance system and improving the speed and efficiency of data collection, analysis, and public health response, WHO in collaboration with the Ministry of Public Health and Population has scaled up the system in all Yemen’s governorates, with sentinel sites expanded from 408 health facilities in 16 governorates to 1186 facilities in 23 governorates after the third expansion at the end of 2016 [3,8].

### Objective

This study is the first evaluation study conducted in Sana’a governorate to identify the strengths and weaknesses of eDEWS, determine its usefulness, and assess its performance in terms of the system attributes, including simplicity, flexibility, data quality, acceptability, representativeness, timeliness, and stability.

## Methods

### Study Design

The CDC’s updated guidelines [9] on the evaluation of public health surveillance were used to evaluate the eDEWS in Sana’a governorate during November to December 2016. Sana’a governorate was covered by eDEWS in 2015. Its population was 918,379 inhabitants, living in 16 districts. The health facilities were at 25 sites in 2015, and with expansion at the end of 2016, health facilities reached 149 sites. Only the first 25 sites were subjected to the evaluation as the expansion happened few months before this evaluation took place.

### Evaluation Approach

To conduct the evaluation, stakeholders from different levels (WHO and Ministry of Public Health & Population (MoPHP), eDEWS central, governorate, and health facility levels) were included. Stakeholders at the central level (national coordinator of eDEWS and staff, including data management officer, data follow-up officer, and the Information Technology (IT) officer), governorate level (coordinator), and health facility level (focal points) in 16 districts were included.

### Data Collection

A total of 4 questionnaires were designed and used to collect data from participants at the 4 levels of eDEWS using face-to-face interview. Each questionnaire comprised items assessing the performance attributes of the system according to the activity of eDEWS at the 4 levels. The assessment of usefulness was limited to governorate, central, and high levels, whereas the assessments of timeliness and completeness were limited only to the central level. Usefulness was assessed by 8 items using 5-point Likert scale (1=strongly disagree, 2=disagree, 3=neutral, 4=agree, and 5=strongly agree) for governorate, central, and high levels. Qualitative and quantitative system attributes were assessed. The qualitative attributes included simplicity, flexibility, acceptability, representativeness, and stability. The quantitative attributes included data quality and timeliness. Weekly reports of eDEWS and available documents of the system were reviewed for completeness and timeliness and to describe the eDEWS surveillance system.

### Data Analysis

The mean percentage scores for usefulness and other performance attributes were calculated by dividing the sum of the items measuring each performance attribute by the maximum score. The scores were interpreted as excellent (>90%), good (80%-90%), average (60%-79.9%), poor (40%-59.9%), and very poor (<40%). The overall attribute score was calculated by summing all items over all attributes and dividing it by the maximum score. During the analysis, the attitude was considered as positive, if the responses were strongly agree or *agree*, whereas the attitude was considered as negative, if the responses were strongly disagree or disagree.

### Ethical Issues

Institution approval for using the program data was obtained from the eDEWS program before beginning the evaluation. Confidentiality of the gathered information was assured by anonymity and using passwords on the computer. Participation was absolutely voluntary.

## Results

### Description of the Electronic Diseases Early Warning System’s Surveillance System

**Figure 1:**
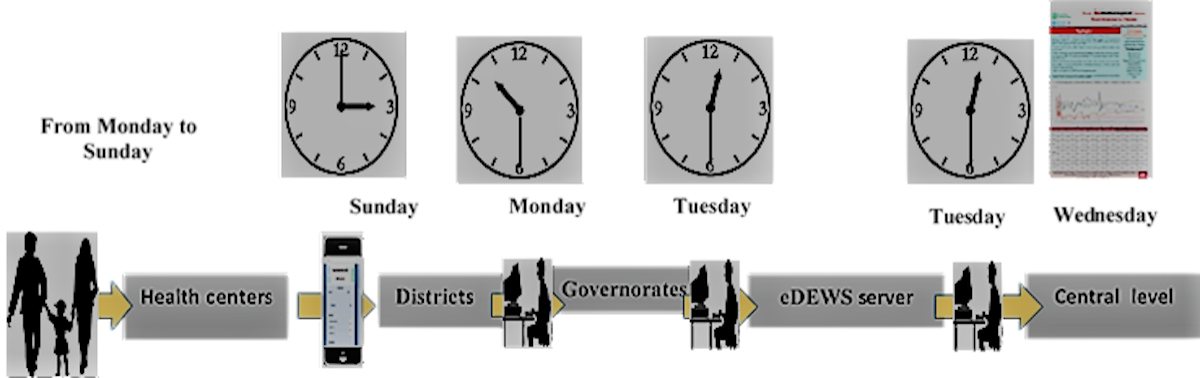
The flow of reports and feedback for the electronic disease early warning system (eDEWS) in Sana’a governorate, Yemen.

The eDEWS is a program in the Diseases Control and Surveillance Directorate. Figure 1 shows the mechanism of the report flow and feedback. eDEWS has 2 main components: (1) immediate alert component for diseases which should be reported within 24 hours after detection such as AFP/polio, cholera, and hemorrhagic fever and (2) weekly reporting component. Weekly data aggregated by the health facilities are reported through the hierarchy of administrative levels (eg, health facility sites, governorates, and central level). At the central level, the system is operated by eDEWS national coordinator, data management officer, IT officer, and data follow-up officer. The program has a governorate coordinator in each governorate and focal point in each health site.

### Usefulness

At the governorate level, the governorate coordinator was asked to appraise the usefulness of eDEWS based on 6 items. The mean percentage was 93% for usefulness. He scored 5 for 5 items and 3 for 1 item. He strongly agreed that the main aim of eDEWS is to detect diseases and outbreaks at an early stage and that data are used to monitor the trends of disease, to estimate the morbidity and mortality for diseases, and to detect outbreaks for rapid action to be taken. However, receiving epidemiological bulletin on time was scored as neutral.

At the high and central levels, the overall score of usefulness was good (mean percentage=83%). All 6 participants strongly agreed that the main aim of eDEWS is the early detection of diseases and outbreaks and all agreed that the data of the system are used to detect morbidity and mortality. All except 1 participant (83.3%) agreed that the data of the system are used to show trends of communicable diseases. In addition, 4 out of 6 (67%) respondents strongly agreed and 2 (33%) agreed that the data are used to facilitate rapid action to be taken.

The governorate coordinator was asked to appraise the simplicity of eDEWS according to 13 items. A total of 8 items were scored 5, 1 item was scored 4, 2 items were scored 3, and 1 item was scored 2. The mean percentage of simplicity was 83%, on which the governorate coordinator strongly agreed that the data entry in the electronic system is easy and clear. In addition, he strongly agreed that the notification process and transferring of data to the central level are easy. The coordinator agreed that the data follow-up is necessary, and analysis of data is conducted with respect to person, place, and time. He also reported that he receives a feedback from the central level. However, he reported that the phone and internet are not always available at his site and training courses are not performed frequently.

### Performance by Attributes

#### Simplicity

The responses of 25 focal points on the simplicity items are shown in Table 1. The simplicity items (15 items) were scored positively by 25 focal points. The overall score of simplicity was good (82%).

#### Acceptability

Table 2 shows the responses of the focal points for each acceptability item (4 items). The overall score of acceptability was 81%, indicating good acceptability.

At the governorate level, the coordinator was asked to appraise the acceptability according to 3 items in which he scored 2 items as 5 and scored 1 item as 3. He strongly agreed that he was willing to support the system and was completely satisfied with eDEWS. However, he was neutral with the program being responsive to his suggestions. The mean percentage of acceptability was 87%.

**Table 1:** The responses of 25 focal points on the items measuring the simplicity of the electronic disease early warning system in Sana’a governorate, Yemen (N=25).

Items	Agree/strongly agree, n (%)	Neutral, n (%)	Disagree/strongly disagree, n (%)
Case definitions for diseases in eDEWS^a^ are available	22 (88)	1 (4)	2 (8)
Case definitions for diseases in eDEWS are easy to apply	22 (88)	2 (8)	1 (4)
Laboratory tests for each disease are always available in your facility	1 (4)	4 (16)	20 (80)
The reporting form is clear to fill	23 (92)	1 (4)	1 (4)
The reporting form is easy to fill	22 (88)	0 (0)	3 (12)
Data entry in the electronic system or phone is clear	22 (88)	2 (8)	1 (4)
Data entry in the electronic system or phone is easy	22 (88)	2 (8)	1 (4)
Notification process is very easy	16 (64)	7 (28)	2 (8)
Phone and internet are always available in your facility	15 (60)	7 (28)	3 (12)
Transferring data to high level is very easy	16 (64)	7 (28)	2 (8)
Data follow-up is necessary to update data on the cases	23 (92)	0 (0)	2 (8)
Involving you in training for eDEWS surveillance	19 (76)	4 (16)	2 (8)
Training courses are performed frequently	20 (80)	4 (16)	1 (4)
The program provided you with phone to facilitate entering and sending of data	22 (88)	3 (12)	0 (0)
Donors provide you with a monthly account to facilitate entering and sending of data	20 (80)	5 (20)	0 (0)

^a^eDEWS: electronic disease early warning system.

**Table 2:** The responses of 25 focal points on the items measuring the acceptability of the electronic disease early warning system in Sana’a governorate, Yemen (N=25).

Items	Agree/strongly agree, n (%)	Neutral, n (%)	Disagree/strongly disagree, n (%)
You are willing to participate in eDEWS^a^ surveillance	24 (96)	1 (4)	0 (0)
You are completely satisfied with eDEWS as a surveillance system	24 (96)	1 (4)	0 (0)
Responsiveness of the system to your suggestions	20 (80)	4 (16)	1 (4)
You receive a feedback report from a governorate level	9 (36)	8 (32)	8 (32)

^a^eDEWS: electronic disease early warning system.

#### Representativeness

At the governorate level, the governorate coordinator was asked to appraise the representativeness of eDEWS based on 2 items; he scored both the items as 5. The mean percentage of representativeness was 100%, in which the participant strongly agreed that the system covered the public and private centers and hospitals (private sites include private hospitals and clinics which have a focal point in each site).

The mean percentage of representativeness in high and central levels was 100%, in which all stakeholders strongly agreed that the system covered the public and private centers and hospitals.

#### Stability

With regard to the stability of eDEWS, the governorate coordinator scored 9 items as follows: 5 for 1 item, 4 for 5 items, 3 for 1 item, and 2 for 2 items. The mean percentage for stability was 71%. The coordinator strongly agreed that there was stable staff, the system was operating in full time, data processing and release were done on a weekly basis, and the electrical power cut rarely occurred because of their own source. He disagreed that the unscheduled outages rarely occurred and that the system would be stable even if the sponsors withdrew their support.

The mean percentage of stability as scored by 6 persons at high and central levels was 69%. All stakeholders agreed that the electrical power cut rarely occurred because of their own source, the staff received monthly incentives to facilitate tasks related to the system, and trained staff were available. A total of 5 out of 6 respondents (83%) were neutral on the unscheduled outage that rarely occurred during the last month. Half of the respondents (50%) disagreed that the system would be stable even if sponsors withdrew their support. All disagreed that there were planned resources for the maintenance of the system.

#### Flexibility

The mean percentage of flexibility reached 91%, in which the 6 stakeholders at high and central levels strongly agreed that the system could accommodate additional information to case definition, could be adapted to integrate with other surveillance programs, and could accommodate new health-related events. A total of 5 out of 6 respondents (83%) agreed that the staff could accommodate changes in data with minimum cost and efforts.

#### Completeness

We checked the completeness of reporting by reviewing the dataset. The reporting rate and completeness during the last 2 months were 100%.

#### Timeliness

According to the responses of the central level, the timeliness of the system was achieved 100%, which was measured as the number of reports that enter the system on time during Sunday evening to Wednesday.

### Summary of the System Performance and Strengths and Weaknesses

The overall system performance was good (86%). The highest attribute score was 100% for representativeness and the lowest score was 70% for stability (Table 3).

**Table 3:** The overall performance of the electronic disease early warning system in Sana’a governorate, Yemen.

Attributes	Health facilities level, %	Governorate level, %	High and central levels, %	Overall percentage, %
Simplicity	82	83	N/A^a^	83
Representativeness	N/A	100	100	100
Acceptability	81	87	N/A	84
Flexibility	N/A	N/A	91	91
Stability	N/A	71	69	70
Overall percentage	82	86	87	86

^a^Not applicable.

All stakeholders at high and central levels were given a chance to express their opinions concerning the strengths and weaknesses of the system. All stakeholders reported that the main strength was the rapid detection and response to diseases and outbreaks. Half of them reported that the data had high accuracy owing to validation that passed through 4 levels and 50% reported that the processing and analysis of data automatically was one of the strengths. On the other hand, all stakeholders in high and central levels said that the main weakness was the network weakness in some remote areas aggravated by war. The director of the system at the central level said that “there is no operational cost for maintenance the electronic instruments and mobiles.”

## Discussion

Our finding revealed that eDEWS is a useful system. This finding is in agreement with the findings of similar evaluations conducted in Sudan [10], Nigeria [11], and Pakistan [12]. However, this finding is not consistent with the eDEWS evaluation that had been conducted in Sana’a city governorate (the capital of Yemen) in 2014, in which the level of usefulness of the system in Sana’a city governorate was poor (54%) [13].

On the basis of the findings of this study, simplicity of the system is good. This finding is in agreement with the findings from previous evaluations in Sana’a city [13] and Madagascar [14].

This study showed that the system had a good acceptability. Similar findings were reported in previous eDEWS evaluations in Sana’a city [13] and in Sudan [10]. The positive appraisal of the system acceptability might be owing to the responsiveness of the program to suggestions and comments of related participants or might be owing to financial support.

The flexibility of the system was found to be excellent. One of the main items of flexibility on which all participants responded positively was the integration of eDEWS with other surveillance programs. Similarly, eDEWS evaluation in Sana’a city [13] and in the Pacific Island countries and territories [15] reported excellent flexibility.

According to the opinions of stakeholders, our evaluation revealed that the stability of the system is average. Poor eDEWS stability was reported in previous evaluation reports in Sana’a city and Madagascar [14]. The low stability found in our study might be explained by that the system relies on the supporting donors completely.

In our evaluation, the completeness was 100% and the reporting rate was 100% in the last 2 months. This finding is in agreement with the finding of a previous eDEWS evaluation in Sana’a city, which revealed that the completeness was 100% [13].

Timeliness in our evaluation received a score of 100%. With regard to timeliness, our finding is nearly in agreement with Sana’a city’s evaluation findings which revealed that the timeliness of the system was 92% in 2014. A study in Madagascar [14] revealed that the timeliness of the system was 68% in 2011.

This evaluation did not include the actual quantitative analysis for some attributes such as positive predictive value and sensitivity. With regard to data quality, accuracy was not assessed owing to the obstacles of comparing the hard reporting forms data with the soft reporting forms data.

In conclusion, our study indicated that eDEWS is a useful system and the overall performance according to the studied attributes was good. As our evaluation was limited to health facilities before the expansion, further evaluation after the expansion is recommended for generalizing the findings on all sites. It is recommended that sending feedback to health facilities focal points should be done in a regular and timely basis (weekly). As the program is completely supported by donors, strengthening the stability of the system by ensuring governmental support is recommended.

## Abbreviations

AFP: Acute Flaccid Paralysis
CDC: Center for Disease Control and Prevention
eDEWS: Electronic Diseases Early Warning System
IT: Information Technology
WHO: World Health Organization
